# Processing of Grammatical Agreement in the Face of Variation in Lexical Stress: A Mismatch Negativity Study

**DOI:** 10.1177/00238309221098116

**Published:** 2022-06-02

**Authors:** Cas W. Coopmans, Marijn E. Struiksma, Peter H. A. Coopmans, Aoju Chen

**Affiliations:** Max Planck Institute for Psycholinguistics, The Netherlands; Centre for Language Studies, Radboud University, The Netherlands; Utrecht Institute of Linguistics OTS, Utrecht University, The Netherlands

**Keywords:** MMN, oddball paradigm, language processing, grammar, subject–verb agreement

## Abstract

Previous electroencephalography studies have yielded evidence for automatic processing of syntax and lexical stress. However, these studies looked at both effects in isolation, limiting their generalizability to everyday language comprehension. In the current study, we investigated automatic processing of grammatical agreement in the face of variation in lexical stress. Using an oddball paradigm, we measured the Mismatch Negativity (MMN) in Dutch-speaking participants while they listened to Dutch subject–verb sequences (linguistic context) or acoustically similar sequences in which the subject was replaced by filtered noise (nonlinguistic context). The verb forms differed in the inflectional suffix, rendering the subject–verb sequences grammatically correct or incorrect, and leading to a difference in the stress pattern of the verb forms. We found that the MMNs were modulated in both the linguistic and nonlinguistic condition, suggesting that the processing load induced by variation in lexical stress can hinder early automatic processing of grammatical agreement. However, as the morphological differences between the verb forms correlated with differences in number of syllables, an interpretation in terms of the prosodic structure of the sequences cannot be ruled out. Future research is needed to determine which of these factors (i.e., lexical stress, syllabic structure) most strongly modulate early syntactic processing.

## 1 Introduction

Language comprehension involves the simultaneous processing of multiple strands of linguistic information, including syntax, lexical stress, and acoustic detail of the speech signal (Friederici, 2011; Hagoort, 2008; Martin, 2016). While these different strands of information can interact even at the earliest stage of processing, they are usually studied in isolation in electrophysiological studies of language processing. For example, Mismatch Negativity (MMN) studies of automatic processing of syntax compare syntactically different sentences that are acoustically and rhythmically similar. Similarly, in MMN studies of automatic processing of lexical stress, a comparison is made between isolated word pairs that have different stress patterns but are otherwise identical. As a result, relatively little is known about what happens when these linguistic factors covary, as occurs in natural language. It is, however, of great importance to examine how linguistic factors interact during language processing, even at the earliest stages of information extraction, as this brings us closer to understanding the natural course of language comprehension in everyday life. Against this background, the current MMN study examines the automatic processing of subject–verb agreement in Dutch when the sequences do not only differ in grammaticality but also in the lexical stress pattern of the verb.

The MMN is an attention-independent negative event-related potential (ERP) effect and is typically elicited by deviant (“oddball”) stimuli in a passive oddball paradigm, in which frequent auditory stimuli (“standards”) are occasionally alternated with infrequent auditory stimuli (“deviants”) (Näätänen et al., 2007). While the MMN was initially considered an index of automatic acoustic change detection (Näätänen & Winkler, 1999), later studies showed that it is also sensitive to higher-order linguistic factors such as syntax and semantics, thus reflecting experience-dependent long-term memory traces (Pulvermüller & Shtyrov, 2006; Shtyrov et al., 2003).

In an MMN study on automatic processing of grammatical agreement, Pulvermüller and Shtyrov (2003) examined the processing of grammatical and ungrammatical English subject–verb sequences (*we come* vs. *we comes) in an oddball paradigm. They hypothesized that syntactic processing takes place automatically via discrete neuronal assemblies that establish binding between categories of grammatically linked lexical items (e.g., pronoun + finite verb). The presentation of a member of the category “pronoun” would thus automatically pre-activate members of the category “verb” in the correctly inflected form. In an oddball paradigm, a pre-activated verb in a correct pronoun-verb sequence (*we come*) was predicted to elicit a smaller MMN than an incorrectly inflected verb, which is not pre-activated by the preceding pronoun (*we comes*). Pulvermüller and Shtyrov (2003) indeed found that the MMN that was triggered by the grammatical deviant was reduced compared with the MMN triggered by the ungrammatical deviant. Interestingly, this difference disappeared when the subject pronoun was replaced by filtered noise (i.e., a sound that was acoustically similar to *we* but unintelligible), showing that the MMN effect reflected automatic processing of syntax. Later studies replicated such “syntactic” MMN effects in other languages (Brunellière et al., 2006, in French; Menning et al., 2005, in German; Shtyrov et al., 2003, in Finnish).

Notably, these studies all manipulated syntactic correctness while keeping prosodic properties of the stimuli constant. One such prosodic property that can covary with syntax and is also subject to automatic processing is lexical stress. In passive oddball experiments, deviant stimuli whose stress pattern differs from that of the standards also elicit an MMN. For example, Weber et al. (2004) looked at the MMN responses to deviants with trochaic stress (strong-weak) presented among standards with iambic stress (weak-strong) and vice versa. The dominant stress pattern in German is trochaic, but iambically stressed words are nevertheless present in German. Weber and colleagues found that, in adults, both deviants elicited a qualitatively similar MMN, regardless of whether they had trochaic or iambic stress (for similar findings in Dutch, see Emmendorfer et al., 2020). In this study, however, it is unclear whether the MMN is triggered by a difference in stress pattern or a difference in the duration of the first syllable (i.e., stressed syllables have a longer duration). In a related study using disyllabic pseudowords in English, a free-stress language, Peter et al. (2012) presented standards which varied in absolute duration but not in the relative duration of the first and the second syllable (e.g., the pattern of the standards in a given block was always short-long). The deviants also varied in absolute duration, but their relative duration pattern was reversed (e.g., long-short). Interestingly, trochaic deviants elicited an MMN, but iambic deviants did not. Because of the variance in the absolute duration of the standards, this difference in MMN is more likely to be driven by the abstract stress pattern of the words than by their acoustic properties. More specifically, Peter et al. (2012) attributed this finding to the listeners’ familiarity with the trochaic stress pattern in English, arguing that the existing memory trace for iambic stress patterns in English listeners might be weaker than that for the trochaic stress pattern, leading to a reduced difference between the ERPs to trochaic standards and iambic deviants and hence a diminished MMN.

The above reviewed studies looked at free-stress languages, in which both trochaic and iambic stress are legal and thus present. This is not the case in languages with a fixed-stress pattern, in which stress always falls on the same syllable. MMN studies in these languages show, among other things, that the processing of lexical stress is influenced by the lexical status of the stress-bearing words. In a study in Finnish, a fixed-stress language with stress on the word-initial syllable, Ylinen et al. (2009) found that the MMN in response to real word deviants with an illegal stress pattern was delayed compared with the MMN in response to real word deviants with the legal stress pattern, suggesting that processing the illegal stress pattern is computationally demanding. However, as the standard stimuli in their experiment were always pseudowords with a legal stress pattern, the response to illegally stressed real word deviants might reflect both lexical and prosodic differences. That lexical familiarity indeed interacts with the processing of lexical stress is suggested by MMN studies in Hungarian, also a fixed-stress language with word-initial stress. When testing pseudowords only, Honbolygó and Csépe (2013) found that the deviant with an illegal stress pattern elicited an MMN, whereas the deviant with a legal stress pattern did not (see also Honbolygó et al., 2020). In contrast, in a follow-up study using real words, MMNs were elicited by both legally and illegally stressed words (Garami et al., 2017).

In sum, past MMN studies have shown that the brain automatically processes information about both syntax and lexical stress. Moreover, the neural response to variation in stress patterns is modulated both by the frequency of those stress patterns in the listener’s native language and by whether the stimuli are real words or pseudowords. It remains unclear, however, whether lexical stress also interacts with syntax at this very early stage of processing, since no study has manipulated both factors in a single experiment.

Such an interaction between prosodic cues and morphosyntactic processing is suggested by a recent series of ERP studies conducted in standard Swedish. In this variety of Swedish, each word has a bitonal lexical pitch accent, so word stem tones constitute strong predictive cues to the upcoming grammatical suffix (Roll et al., 2013; Söderström, Horne, & Roll, 2017a). These studies found that when the word stem tone incorrectly cued a suffix, the suffix elicited a P600 effect, sometimes preceded by a Left Anterior Negativity (Roll et al., 2010, 2013; Söderström et al., 2017a, 2017b). This Left Anterior Negativity was taken as an indicator of morphosyntactic processing, possibly reflecting the activation of an unprimed memory trace of a suffix (Söderström et al., 2017a). In light of these findings, the authors argued that listeners can use neural tone–suffix connections to rapidly pre-activate upcoming suffixes based on tonal information. It remains to be investigated whether automatic processing of lexical stress also influences early syntactic processing, in particular in a language in which these strands of information are not predictively cued by one another.

### 1.1 The present study

The foregoing literature review reveals two main findings. First, manipulations of both grammatical agreement and lexical stress independently modulate MMN amplitude, indicating that syntactic and prosodic information are both processed automatically at a very early stage in the processing stream. Second, prosodic information affects subsequent processing of morphosyntactic information, as evidenced by a modulation of the LAN and the P600. In the present study, we investigate whether and how variations of lexical stress modulate automatic processing of grammatical agreement as indexed by the MMN.

We adapted Pulvermüller and Shtyrov’s (2003) oddball paradigm by introducing an additional difference between the grammatical subject–verb sequences (*wij dansen* “we dance”) and their ungrammatical counterparts (*wij danst “we dances”), that is, the stress pattern of the verb. The plural form of the verb *dansen* is disyllabic and the stress falls on the penultimate syllable, whereas the singular form of the verb *danst* is monosyllabic and has no penultimate stress. Penultimate stress in disyllabic words results in trochaic stress, the dominant stress pattern in Dutch, a free-stress language (Gussenhoven, 2009). In general, the plural form of finite verbs in Dutch is created by adding the suffix -*en* to the word stem, making these forms multisyllabic. Hence, finite verbs that are preceded by *wij* are typically multisyllabic with penultimate stress.

The findings reviewed above suggest two competing hypotheses. On one hand, in line with Pulvermüller and Shtyrov (2003) and similar studies, we hypothesize that automatic processing of subject–verb agreement proceeds independently of the processing of lexical stress. We predict that the MMN in response to the grammatical deviant (*wij dansen*) will be smaller than the MMN to the ungrammatical deviant (*wij danst) and that there will be no such difference in the nonlinguistic context condition. On the other hand, in light of existent evidence for an early effect of prosodic information on morphosyntactic processing, we hypothesize that the processing of stress-related information may interact with automatic processing of subject–verb agreement. Because of Dutch speakers’ familiarity with the trochaic stress pattern of *dansen*, we predict that the MMN to deviants with *dansen* as the verb will be larger than the MMN to deviants with *danst* as the verb (Peter et al., 2012). While we expect this difference to be present in both the linguistic and the nonlinguistic condition, it might be smaller in the linguistic condition because of the additional automatic processing of subject–verb agreement. Note that the verb forms also differ segmentally after the stem *dans*. Although *dans* is followed by additional phonemes in both verb forms, the phonemes are different: /ən/ in *dansen* vs. /t/ in *danst*. Because the verb forms in past studies on automatic grammar processing have comparable acoustic differences in the suffix (Brunellière et al., 2006; Menning et al., 2005; Pulvermüller & Shtyrov, 2003; Shtyrov et al., 2003), we assume that such phonetic differences will not have the same impact on the processing of the pronoun–verb agreement as phonological differences such as a difference in the lexical stress pattern. This point will be revisited in the discussion section.

## 2 Methods

### 2.1 Participants

Twenty-nine right-handed native speakers of Dutch participated in the experiment (18–29 years; mean age = 22 years; 21 females). Written consent was obtained from all participants after they had been informed about the study, along with confirmation that they had no hearing deficits and were not dyslectic. The participants received a fee for their participation.

### 2.2 Stimuli

**Figure 1.**
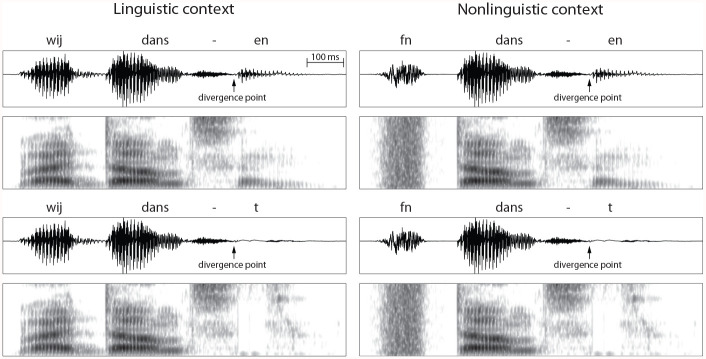
Acoustic waveforms and corresponding spectrograms of the four stimuli used in the experiment. The verbs were identical up to the divergence point, which was placed between the verb stem (*dans*-) and the inflection (-*en* or -*t*). The acronym *fn* stands for filtered noise, which has the acoustic properties of the pronoun *wij* but is unintelligible. The onset of *wij/fn* was preceded by a 50 ms silence; the offset of the verb was followed by a 100 ms silence. See Supplementary Material for acoustic details of the stimuli.

Two Dutch subject–verb sequences were used as stimuli, consisting of a personal pronoun and a verb. The personal pronoun *wij* was either followed by the grammatically correct verb form *dansen* “we dance” or by the grammatically incorrect *danst* “*we dances”. In the experimental (linguistic) condition, the personal pronoun provided the linguistic context to the verb. In the control (nonlinguistic) condition, the verb was always preceded by a nonlinguistic sound, which had acoustic characteristics similar to those of the personal pronoun but was not intelligible (see Figure 1). We included this nonlinguistic condition to control for acoustic and metrical differences between *wij dansen* and *wij danst* that might affect the mismatch response. If the difference in MMNs to these stimuli reflects processing of lexical stress, it should also be found in the nonlinguistic condition, because the nonlinguistic sound has the acoustic properties of the pronoun and because the stress patterns of the linguistic and nonlinguistic sequences are identical. In contrast, if the difference reflects processing of grammatical agreement, it should not be found in response to verbs that are preceded by a nonlinguistic sound instead of a pronoun.

A male native speaker of Dutch recorded the two pronoun–verb combinations at a sampling rate of 44.1 kHz and a resolution of 16 Bit. The most clearly articulated phrases were selected and subjected to speech manipulation in Praat (Boersma & Weenink, 2014) to ensure that both types of pronoun–verb combinations were acoustically identical up to the crucial point of grammatical deviation (i.e., divergence point). Specifically, the verb stem of *dansen* (*dans-*) was used for both the grammatical and ungrammatical sequence because it was easier to separate *dans* from *dansen* than from *danst* due to a larger degree of coarticulation in the latter. The *wij danst* stimulus was created by replacing the suffix -*en* from *dansen* with the suffix -*t* from *danst.* The originally recorded *wij dansen* stimulus was used as the grammatical stimulus. Both *wij dansen* and the concatenated *wij danst* sounded natural to native speakers of Dutch.

The nonlinguistic sound, hereafter referred to as *fn* (*f*iltered *n*oise), was created in Praat (Boersma & Weenink, 2014) via the following procedure. First, the pitch and intensity contours were extracted from the pronoun *wij* in the original recordings by means of Linear Predictive Coding. Second, Gaussian white noise was synthesized, which was filtered by the pitch contour and multiplied by the intensity contour of the pronoun. As a result, the white noise had the same loudness profile and natural intonation as the pronoun in the linguistic condition. Finally, the average intensity of the nonlinguistic sound was scaled to 65 dB, an arbitrarily selected value that resulted in a consistent volume in perception across the filtered noise and the natural speech components of the stimulus.

### 2.3 Design

An auditory passive oddball paradigm was used, in which each of the four stimulus sequences appeared once as the deviant and once as the standard stimulus. Each standard–deviant combination was presented in a block of 250 stimuli (210 repetitions [84%] of standard and 40 repetitions [16%] of deviant). A total of 1,000 stimuli were presented to the participants. In block 1, *fn danst* was the deviant and *fn dansen* the standard, while the opposite was the case in block 2. In block 3, *wij danst* was the deviant and *wij dansen* the standard, while the reverse order was used in block 4. The order of the blocks was partially counterbalanced, such that blocks with *dansen* as deviant preceded blocks with *danst* as deviant equally often as vice versa. Standards and deviants were presented in a pseudorandom order with an average stimulus onset asynchrony of 1,400 ms. A slightly varying interstimulus interval (between 11 and 55 ms) was used to keep the amount of externally generated noise (i.e., electromagnetic noise originating from electronic devices) in the signal low. The experiment was conducted using the Presentation software (Version 18.1).

### 2.4 Procedure

This study was conducted following the ethical guidelines of the Utrecht University Institute of Linguistics. Each participant was tested individually. They were seated in front of a computer screen and watched a silent movie while the stimuli were auditorily presented over Tangent Evo E4 audio speakers, which were placed approximately 70 cm from the participant. They were instructed to watch the video and ignore the auditory signals to minimize the possibility that they would consciously and strategically approach the stimuli. The electroencephalogram (EEG) was recorded using 64 Ag/AgCl electrode BioSemi caps with standardized 10–20 configuration and a BioSemi ActiveTwo system at a sampling rate of 2048 Hz. We used two additional electrodes at the mastoids for referencing and six bipolar electro-oculogram electrodes to record horizontal and vertical eye movements. The recording session lasted 1 hour on average.

### 2.5 EEG processing

Preprocessing of the EEG data was performed in BrainVision Analyzer (version 2.1; Brain Products, Munich, Germany). The data were re-referenced offline to the average of the two mastoids, band-pass filtered at 0.1–35 Hz (24 dB/oct) and downsampled to 500 Hz. Epochs ranging from –200 to 700 ms were extracted relative to the divergence point, which was chosen as the zero point because the standard and deviant in each block were identical up to that point.^1^ ERPs were normalized to a 200 ms baseline window preceding the divergence point. We used an Ocular Correction transform based on Independent Component Analysis to filter artifacts resulting from eye blinks (vEOG) and eye movements (hEOG). Finally, we rejected individual channel-segment pairs which contained artifacts with an amplitude exceeding ± 75 μV, displaying a voltage step of 50 μV or more between two neighboring sampling points, or in which the difference in signal activity was lower than 0.5 μV in an interval of 100 ms (excluding <1% of the data).

## 3 Results

For each channel, average MMNs were computed by subtracting, for each unique stimulus, the ERP response to the standard from the ERP response to the deviant.^2^ For example, the MMN to *wij dansen* refers to the difference wave resulting from *wij dansen* presented as deviant minus *wij dansen* as standard.

**Figure 2.**
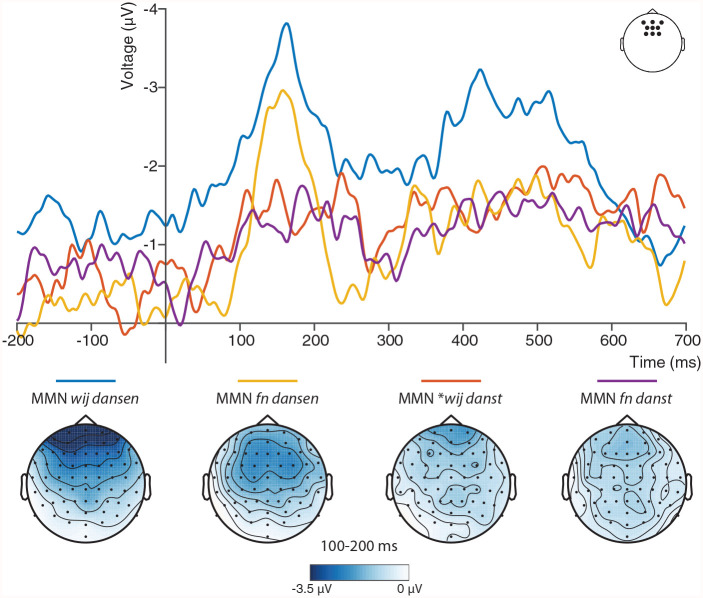
Grand average ERPs in the frontal region of interest for the Mismatch Negativities (MMNs) to *wij*/*fn dansen/danst*, computed as deviant minus standard (negative voltage is plotted upward). Scalp distributions of each MMN in the 100–200 ms time window are plotted below.

Given that the MMN peaks around 150 ms after deviance onset and has a frontal distribution, we performed statistical analysis on the average activity within a frontal region of interest (frontal electrodes FCz, FC1, FC2, Fz, F1, F2, AFz, AF3, and AF4) and within a time window ranging from 100 to 200 ms after deviance onset. A repeated measures ANOVA was conducted to assess the effects of Form (*dansen* vs. *danst*) and Context (*wij* vs. *fn*) on MMN amplitude. This analysis revealed a main effect of Form, such that the MMN to *dansen* was significantly larger than the MMN to *danst, F*(1, 28) = 8.74, *p* = .006, η^2^ = 0.24 (see Figure 2).^3^ We did not find a main effect of Context, *F*(1, 28) = 0.61, *p* = .44, nor an interaction of Form by Context, *F*(1, 28) = 1.00, *p* = .33.^4^

Given the absence of an effect of linguistic context in the hypothesis-driven spatiotemporal region of interest (ROI), we further explored whether there were any effects beyond this specific ROI. As an exploratory analysis, we ran a cluster-based permutation test (Maris & Oostenveld, 2007) in the entire 0–700 ms time window and across all electrodes, testing the effects of Context, Form, as well as their interaction (i.e., difference between the MMNs to *dansen* and *danst* in the linguistic vs. the nonlinguistic context). Effects were compared to a reference distribution based on 1,000 random permutations of the same data and were considered significant if their cluster-level *p* value was below α = 0.05. In line with the MMN effect reported above, these three analyses yielded only an effect of Form: the *dansen* stimuli elicited a more negative ERP signal than the *danst* stimuli (one significant negative cluster, *p* = .03). This effect was most prominent between roughly 120 and 190 ms after the divergence point and had a widely distributed frontal topography.

## 4 Discussion

In this EEG study, we aimed to find out whether lexical stress affects automatic processing of grammatical agreement, as indicated by modulations of the Mismatch Negativity (MMN). We compared MMNs to deviants that were grammatical and had the typical stress pattern in Dutch for a sequence of plural pronoun and verb (*wij/fn dansen*) to MMNs to deviants that were ungrammatical and had a nontypical stress pattern (*wij/fn danst*). We found that the MMN in response to *dansen* was larger than the MMN in response to *danst*, regardless of the context provided by the pronoun (main effect of Form). The complete absence of an effect of Context (i.e., no difference between the MMNs based on whether the verbs were preceded by *wij* or *fn*) suggests that the processing load induced by modulations of lexical stress can hinder automatic processing of grammatical agreement.

Previous ERP studies have shown that metrical variation affects syntactic processing at a later stage of processing, as evidenced by the fact that the P600 response to syntactically difficult structures is modulated by metrical violations (Schmidt-Kassow & Kotz, 2008 ) and by differences in rhythmic regularity (Roncaglia-Denissen et al., 2013). The P600 is often interpreted as reflecting a late stage in the processing stream where multiple sources of linguistic information are integrated (Kaan & Swaab, 2003) and is highly sensitive to task demands, such as the presence of a judgment task (Kolk et al., 2003; Schacht et al., 2014). Our findings suggest that lexical stress can affect syntactic processing even at a very early stage of information extraction when the participants do not pay attention to the stimuli.

It should be noted that our results do not indicate that lexical stress interacted with grammatical agreement, but rather that the latter might be insufficiently processed due to the variation in lexical stress. If the processing load induced by prosodic information is the same in the deviant as in the standard, as was the case in previous syntactic MMN studies (Brunellière et al., 2006; Menning et al., 2005; Pulvermüller & Shtyrov, 2003; Pulvermüller et al., 2008; Shtyrov et al., 2003), processing of grammatical agreement might be unaffected. In contrast, when the deviant has a stress pattern different from the standard, as in our study, this may dominate the processing, leaving no resources for automatic processing of grammatical agreement at the same time. This may especially be the case in a passive listening MMN paradigm, where attention is distracted from the input and processing resources are limited.

Although we interpret our findings as an effect of variation in lexical stress in the stimuli, there is an important caveat to this interpretation. The metrical difference between the verb forms is confounded by a phonological difference that we initially did not consider but that was brought to our attention during the review process. That is, the verb forms also differ in syllabic structure or number of syllables: the plural form *dansen* is disyllabic, the third-person singular form *danst* is monosyllabic. The speculated metrical difference between *dansen* and *danst* can thus also be considered in the first place a difference in number of syllables, which causes the difference in the prosodic structure of the subject–verb sequence. Although past work suggests that the processing of low-level segmental differences such as the difference between *come* and *comes* does not interfere with the processing of grammatical agreement (Brunellière et al., 2006; Menning et al., 2005; Pulvermüller & Shtyrov, 2003; Shtyrov et al., 2003), it remains to be investigated whether the same applies to phonological differences between verb forms such as syllabic structure. Future research on other languages or artificial languages using subject–verb agreement constructions that differ only in stress pattern, or on Dutch using other types of syntactic constructions, is needed to validate our results. As an example of such a stimulus type, the Dutch two-word combination *het onderzoek* can be a noun phrase or a finite verb phrase, depending on the stress pattern of the second word (i.e., *het onderzoek* “the investigation” vs. *het onderzoek* “investigate it”). The combination of *het* *onderzoek* can, however, only occur in very restricted contexts and is not considered grammatical on its own. It is thus much more common to hear the noun *onderzoek* than the first-person singular finite verb form *onderzoek* after the word *het.*

An alternative way to interpret our results is to look at processing at a level higher than the verb forms. The presence of an effect of Form indicates that, in both a linguistic and a nonlinguistic context, processing *dansen* after repetitions of *danst* is more demanding than processing *danst* after repetitions of *dansen*. This may suggest that the prosodic structure of the entire context (i.e., the sequence of standards) affects the processing of the deviant stimulus. The MMN in an oddball design might thus reflect not only a response to the form of the deviant itself but also a response to the commonality of the form of the entire sequence of standards and deviant in the target language. This idea is compatible with findings from research on the effects of long-term memory representations on the processing of stress patterns. For instance, Kriukova and Mani (2016) used an oddball-like design to examine ERP responses of native speakers of German while they silently read sequences of disyllabic German words. Both the stress pattern of the last word (trochee vs. iamb) and the metrical congruence between the last word and the preceding three words were manipulated. The iambic critical words elicited an attenuated negativity around 250–400 ms in the incongruent condition (trochee-trochee-trochee-*iamb*) compared with the congruent condition (iamb-iamb-iamb-*iamb*), whereas there were no effects of congruency on ERP responses to trochaic sequence-final words. Considering that the typical metrical pattern in German contains occasional iambs alternating with frequent trochees, Kriukova and Mani (2016) argued that processing sequences of trochee-trochee-trochee-*iamb* is less demanding than processing sequences in which the iambic sequence-final word is preceded by only iambs. Similarly, in the present study, the sequence in which the deviant *danst* was preceded by multiple repetitions of *dansen* reflects the typical metrical pattern of Dutch. More specifically, although the stress pattern and the syllabic structure of the deviant *danst* do not match with a short-term memory trace formed on the basis of multiple repetitions of *dansen*, the metrical pattern of that entire sequence of standards and deviants is similar to the listener’s long-term memory trace of rhythmic alternation in Dutch. Thus, when preceded by multiple repetitions of *wij dansen* (weak-strong-weak or wsw), the sequence *wij danst* may not be more unexpected than the sequence *wij dansen*. Processing of *wij danst* in this situation might thus be facilitated compared with the reverse situation, in which the sequence *wij dansen* is preceded by multiple repetitions of *wij danst* (ws-ws-ws-wsw). The latter situation is less like the typical metrical pattern in Dutch, and does not facilitate processing of *wij dansen*. Our finding thus provides additional evidence that the MMN can reflect both short-term memory representations about the stress pattern of the previous stimuli and long-term memory representations about the rhythm of one’s native language (Näätänen, 2001; Näätänen et al., 2007; Winkler et al., 1996).

## 5 Conclusions

While more conclusive evidence for the influence of the processing of lexical stress on early processing of grammatical agreement awaits further research, this study shows that early processing of grammatical agreement is affected by the processing of phonological properties of the speech signal. This casts doubt on the generalizability of previous MMN studies, which have studied the processing of different strands of information in isolation. Further research on how early processing of information affects processing at later stages is of vital importance to gaining better understanding of the natural course of language comprehension in everyday life.

## Supplemental Material

sj-docx-1-las-10.1177_00238309221098116 – Supplemental material for Processing of Grammatical Agreement in the Face of Variation in Lexical Stress: A Mismatch Negativity Study Click here for additional data file. Supplemental material, sj-docx-1-las-10.1177_00238309221098116 for Processing of Grammatical Agreement in the Face of Variation in Lexical Stress: A Mismatch Negativity Study by Cas W. Coopmans, Marijn E. Struiksma, Peter H. A. Coopmans and Aoju Chen in Language and Speech

## Notes

1. The inflection in the linguistic and nonlinguistic condition started after 632 ms and 612 ms, respectively, which was due to the fact that the interval between the onset of *wij/fn* and the onset of the verb was 20 ms longer in the linguistic than in the nonlinguistic condition. This did not affect any perceived mismatch responses, given that the stimuli presented in the same block always had the same duration.

2. The data of this study are publicly available via our OSF page: https://osf.io/ksqpn/.

3. We note that the baseline of the MMN to *wij dansen* is slightly higher than the baseline of the other three MMNs, yielding an elevated ERP in the 100–200 ms time window. Applying an additional baseline correction to the grand average ERPs yielded MMNs with similar baselines, and supports the main effect of Form (see Supplementary Material).

4. For the sake of completeness, we also compared the conditions in the two time windows in which Pulvermüller and Shtyrov (2003) found grammaticality effects. The analysis in the 130–170 ms time window shows a main effect of Form, *F*(1, 28) = 16.6, *p* < .001, η^2^ = 0.37, but no effect of Context, *F*(1, 28) = 0.68, *p* = .42, and no interaction, *F*(1, 28) = 0.06, *p* = .82. Repeating this analysis in the 330–370 ms time window does not lead to any significant effects: no effect of Form, *F*(1, 28) = 0.61, *p* = .0.44, no effect of Context, *F*(1, 28) = 0.61, *p* = .41, and no interaction, *F*(1, 28) = 0.03, *p* = .87.

## References

[bibr1-00238309221098116] Boersma P. Weenink D. (2014). Praat: Doing phonetics by computer (Version 5.4.01) [Computer Program]. https://www.praat.org/

[bibr2-00238309221098116] Brunellière A. Franck J. Ludwig C. Frauenfelder U. H. (2006). Early and automatic syntactic processing of person agreement. NeuroReport, 18, 537–541. 10.1097/WNR.0b013e3280b07ba1 17413653

[bibr3-00238309221098116] Emmendorfer A. K. Correia J. M. Jansma B. M. Kotz S. A. Bonte M. (2020). ERP mismatch response to phonological and temporal regularities in speech. Scientific Reports, 10(1), Article 9917. 10.1038/s41598-020-66824-x PMC7303198 32555256

[bibr4-00238309221098116] Friederici A. D. (2011). The brain basis of language processing: From structure to function. Physiological Reviews, 91, 1357–1392. 10.1152/physrev.00006.2011 22013214

[bibr5-00238309221098116] Garami L. Ragó A. Honbolygó F. Csépe V. (2017). Lexical influence on stress processing in a fixed-stress language. International Journal of Psychophysiology, 117, 10–16. 10.1016/j.ijpsycho.2017.03.006 28377265

[bibr6-00238309221098116] Gussenhoven C. (2009). Vowel duration, syllable quantity, and stress in Dutch. In Hanson K. Inkelas S. (Eds.), The nature of the word. Essays in honor of Paul Kiparsky (pp. 181–198). MIT Press.

[bibr7-00238309221098116] Hagoort P. (2008). The fractionation of spoken language understanding by measuring electrical and magnetic brain signals. Philosophical Transactions of the Royal Society B: Biological Sciences, 363(1493), 1055–1069. 10.1098/rstb.2007.2159 PMC2606796 17890190

[bibr8-00238309221098116] Honbolygó F. Csépe V. (2013). Saliency or template? ERP evidence for long-term representation of word stress. International Journal of Psychophysiology, 87(2), 165–172. 10.1016/j.ijpsycho.2012.12.005 23275150

[bibr9-00238309221098116] Honbolygó F. Kóbor A. German B. Csépe V. (2020). Word stress representations are language-specific: Evidence from event-related brain potentials. Psychophysiology, 57(5), e13541. 10.1111/psyp.13541 32022278

[bibr10-00238309221098116] Kaan E. Swaab T. Y. (2003). Repair, revision, and complexity in syntactic analysis: An electrophysiological differentiation. Journal of Cognitive Neuroscience, 15, 98–110. 10.1162/089892903321107855 12590846

[bibr11-00238309221098116] Kolk H. H. Chwilla D. J. Van Herten M. Oor P. J. (2003). Structure and limited capacity in verbal working memory: A study with event-related potentials. Brain and Language, 85, 1–36. 10.1016/S0093-934X(02)00548-5 12681346

[bibr12-00238309221098116] Kriukova O. Mani N. (2016). Processing metrical information in silent reading: An ERP study. Frontiers in Psychology, 7, Article 1432. 10.3389/fpsyg.2016.01432 PMC5031776 27713718

[bibr13-00238309221098116] Maris E. Oostenveld R. (2007). Nonparametric statistical testing of EEG- and MEG-data. Journal of Neuroscience Methods, 164(1), 177–190. 10.1016/j.jneumeth.2007.03.024 17517438

[bibr14-00238309221098116] Martin A. E. (2016). Language processing as cue integration: Grounding the psychology of language in perception and neurophysiology. Frontiers in Psychology, 7, Article 120. 10.3389/fpsyg.2016.00120 PMC4754405 26909051

[bibr15-00238309221098116] Menning H. Zwitserlood P. Schöning S. Hihn H. Bölte J. Dobel C. Mathiak K. Lütkenhöner B. (2005). Pre-attentive detection of syntactic and semantic errors. NeuroReport, 16, 77–80. 10.1097/00001756-200501190-00018 15618895

[bibr16-00238309221098116] Näätänen R. (2001). The perception of speech sounds by the human brain as reflected by the mismatch negativity (MMN) and its magnetic equivalent (MMNm). Psychophysiology, 38, 1–21. 10.1111/1469-8986.3810001 11321610

[bibr17-00238309221098116] Näätänen R. Paavilainen P. Rinne T. Alho K. (2007). The mismatch negativity (MMN) in basic research of central auditory processing: A review. Clinical Neurophysiology, 118, 2544–2590. 10.1016/j.clinph.2007.04.026 17931964

[bibr18-00238309221098116] Näätänen R. Winkler I. (1999). The concept of auditory stimulus representation in cognitive neuroscience. Psychological Bulletin, 125, 826–859. 10.1037/0033-2909.125.6.826 10589304

[bibr19-00238309221098116] Peter V. McArthur G. Thompson W. F. (2012). Discrimination of stress in speech and music: A mismatch negativity (MMN) study. Psychophysiology, 49, 1590–1600. 10.1111/j.1469-8986.2012.01472.x 23066846

[bibr20-00238309221098116] Pulvermüller F. Shtyrov Y. (2003). Automatic processing of grammar in the human brain as revealed by the mismatch negativity. NeuroImage, 20, 159–172. 10.1016/S1053-8119(03)00261-1 14527578

[bibr21-00238309221098116] Pulvermüller F. Shtyrov Y. (2006). Language outside the focus of attention: The mismatch negativity as a tool for studying higher cognitive processes. Progress in Neurobiology, 79, 49–71. 10.1016/j.pneurobio.2006.04.004 16814448

[bibr22-00238309221098116] Pulvermüller F. Shtyrov Y. Hasting A. S. Carlyon R. P. (2008). Syntax as a reflex: Neurophysiological evidence for early automaticity of grammatical processing. Brain and Language, 104(3), 244–253. 10.1016/j.bandl.2007.05.002 17624417

[bibr23-00238309221098116] Roll M. Horne M. Lindgren M. (2010). Word accents and morphology—ERPs of Swedish word processing. Brain Research, 1330, 114–123. 10.1016/j.brainres.2010.03.020 20298679

[bibr24-00238309221098116] Roll M. Söderström P. Horne M. (2013). Word stem tones cue suffixes in the brain. Brain Research, 1520, 116–120. 10.1016/j.brainres.2013.05.013 23685193

[bibr25-00238309221098116] Roncaglia-Denissen M. P. Schmidt-Kassow M. Kotz S. A. (2013). Speech rhythm facilitates syntactic ambiguity resolution: ERP evidence. PLOS ONE, 8, Article e56000. 10.1371/journal.pone.0056000 PMC3568096 23409109

[bibr26-00238309221098116] Schacht A. Sommer W. Shmuilovich O. Martíenz P. C. Martín-Loeches M. (2014). Differential task effects on N400 and P600 elicited by semantic and syntactic violations. PLOS ONE, 9, Article e91226. 10.1371/journal.pone.0091226 PMC3948820 24614675

[bibr27-00238309221098116] Schmidt-Kassow M. Kotz S. A. (2008). Event-related brain potentials suggest a later interaction of meter and syntax in the P600. Journal of Cognitive Neuroscience, 21, 1693–1708. 10.1162/jocn.2008.21153 18855546

[bibr28-00238309221098116] Shtyrov Y. Pulvermüller F. Näätänen R. Ilmoniemi R. J. (2003). Grammar processing outside the focus of attention: An MEG study. Journal of Cognitive Neuroscience, 15, 1195–1206. 10.1162/089892903322598148 14709236

[bibr29-00238309221098116] Söderström P. Horne M. Mannfolk P. van Westen D. Roll M. (2017b). Tone-grammar association within words: Concurrent ERP and fMRI show rapid neural pre-activation and involvement of left inferior frontal gyrus in pseudoword processing. Brain and Language, 174, 119–126. 10.1016/j.bandl.2017.08.004 28850882

[bibr30-00238309221098116] Söderström P. Horne M. Roll M. (2017a). Stem tones pre-activate suffixes in the brain. Journal of Psycholinguistic Research, 46, 271–280. 10.1007/s10936-016-9434-2 27240896 PMC5368231

[bibr31-00238309221098116] Weber C. Hahne A. Friedric M. Friederici A. D. (2004). Discrimination of word stress in early infant perception: Electrophysiological evidence. Cognitive Brain Research, 18, 149–161. 10.1016/j.cogbrainres.2003.10.001 14736574

[bibr32-00238309221098116] Winkler I. Cowan N. Csépe V. Czigler I. Näätänen R. (1996). Interactions between transient and long-term auditory memory as reflected by the mismatch negativity. Journal of Cognitive Neuroscience, 8, 403–415. 10.1162/jocn.1996.8.5.403 23961944

[bibr33-00238309221098116] Ylinen S. Strelnikov K. Huotilainen M. Näätänen R. (2009). Effects of prosodic familiarity of the automatic processing of words in the human brain. International Journal of Psychophysiology, 733, 362–368. 10.1016/j.ijpsycho.2009.05.013 19482051

