# Supplementary material for: Processing of Grammatical Agreement in the Face of Variation in Lexical Stress: A Mismatch Negativity Study
Source: Lang Speech. 2022 Jun 2;66(1):202–13. doi: 10.1177/00238309221098116 (PMC9976639; doi:10.1177/00238309221098116)
Supplement: sj-docx-1-las-10.1177_00238309221098116 – Supplemental material for Processing of Grammatical Agreement in the Face of Variation in Lexical Stress: A Mismatch Negativity Study [file sj-docx-1-las-10.1177_00238309221098116.docx]

**Supplementary Material**

**1. Acoustic measurements of the stimuli**

**Table 1.** Acoustic measurements of the stimulus ‘wij dansen’.

|  | wij | dans- | en | wij dansen |
| --- | --- | --- | --- | --- |
| duration (ms) | 227 | 355 | 193 | 775 |
| mean pitch (Hz) | 97 | 100 | |  |
| pitch-minimum (Hz) | 97 | 92 | |  |
| pitch-maximum (Hz) | 103 | 114 | |  |
| mean intensity (dB) |  |  |  | 70 |

**Table 2.** Acoustic measurements of the stimulus ‘wij danst’.

|  | wij | dans- | t | wij danst |
| --- | --- | --- | --- | --- |
| duration (ms) | 227 | 355 | 193 | 775 |
| mean pitch (Hz) | 97 | 100 | |  |
| pitch-minimum (Hz) | 97 | 92 | |  |
| pitch-maximum (Hz) | 103 | 114 | |  |
| mean intensity (dB) |  |  |  | 70 |

**Table 3.** Acoustic measurements of the stimulus ‘fn dansen’.

|  | fn | dans- | en | fn dansen |
| --- | --- | --- | --- | --- |
| duration (ms) | 207 | 355 | 193 | 755 |
| mean pitch (Hz) |  | 100 | |  |
| pitch-minimum (Hz) |  | 92 | |  |
| pitch-maximum (Hz) |  | 114 | |  |
| mean intensity (dB) |  |  |  | 70 |

**Table 4.** Acoustic measurements of the stimulus ‘fn danst’.

|  | fn | dans- | t | fn danst |
| --- | --- | --- | --- | --- |
| duration (ms) | 207 | 355 | 193 | 755 |
| mean pitch (Hz) |  | 106 | |  |
| pitch-minimum (Hz) |  | 102 | |  |
| pitch-maximum (Hz) |  | 114 | |  |
| mean intensity (dB) |  |  |  | 70 |

**2. Additional baseline correction**

The baseline of the Mismatch Negativity (MMN) to *wij* *dansen* seemed slightly higher than the baseline of the other three MMNs, resulting in an elevated MMN within the 100-200 ms time window. Yet, the interaction was not significant. Applying an additional baseline correction to the grand average ERPs yielded MMNs with similar baselines (see Figure 1). The analysis reported in the text is in line with both figures and supports the main effect of Form (i.e. larger MMN to *dansen* than to *danst*).


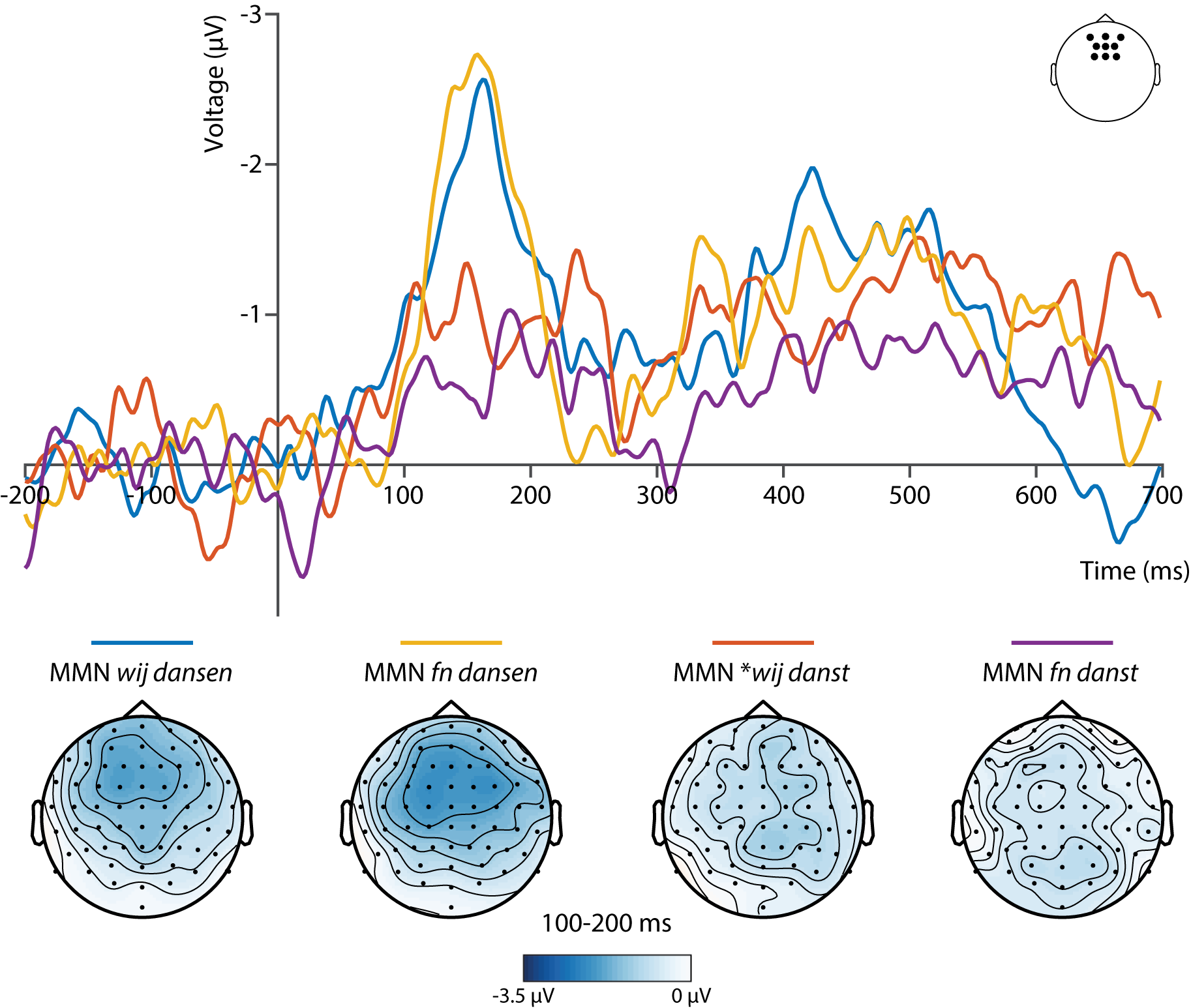


**Figure 1.** Main MMN results with an additional baseline correction. Grand average ERPs of the frontal region of interest for the MMNs to *wij*/*fn* *dansen/danst*, computed as deviant minus standard (negative voltage is plotted upwards), with an additional baseline correction. Scalp distributions of each MMN in the 100-200 ms time window are plotted below.
